# Non-Additive Entropic Forms and Evolution Equations for Continuous and Discrete Probabilities

**DOI:** 10.3390/e25081132

**Published:** 2023-07-27

**Authors:** Evaldo M. F. Curado, Fernando D. Nobre

**Affiliations:** Centro Brasileiro de Pesquisas Físicas and National Institute of Science and Technology for Complex Systems Rua Xavier Sigaud 150, Urca, Rio de Janeiro 22290-180, Brazil

**Keywords:** nonlinear Fokker–Planck equations, generalized entropies, nonextensive thermostatistics, 05.70.Ln, 05.40.Fb, 05.90.+m, 05.10.Gg, 05.20.-y

## Abstract

Increasing interest has been shown in the subject of non-additive entropic forms during recent years, which has essentially been due to their potential applications in the area of complex systems. Based on the fact that a given entropic form should depend only on a set of probabilities, its time evolution is directly related to the evolution of these probabilities. In the present work, we discuss some basic aspects related to non-additive entropies considering their time evolution in the cases of continuous and discrete probabilities, for which nonlinear forms of Fokker–Planck and master equations are considered, respectively. For continuous probabilities, we discuss an H-theorem, which is proven by connecting functionals that appear in a nonlinear Fokker–Planck equation with a general entropic form. This theorem ensures that the stationary-state solution of the Fokker–Planck equation coincides with the equilibrium solution that emerges from the extremization of the entropic form. At equilibrium, we show that a Carnot cycle holds for a general entropic form under standard thermodynamic requirements. In the case of discrete probabilities, we also prove an H-theorem considering the time evolution of probabilities described by a master equation. The stationary-state solution that comes from the master equation is shown to coincide with the equilibrium solution that emerges from the extremization of the entropic form. For this case, we also discuss how the third law of thermodynamics applies to equilibrium non-additive entropic forms in general. The physical consequences related to the fact that the equilibrium-state distributions, which are obtained from the corresponding evolution equations (for both continuous and discrete probabilities), coincide with those obtained from the extremization of the entropic form, the restrictions for the validity of a Carnot cycle, and an appropriate formulation of the third law of thermodynamics for general entropic forms are discussed.

## 1. Introduction

The area of complex systems has attracted the attention of many researchers in recent years and has exhibited a large variety of novel phenomena, such as nonlinear dynamics, slow relaxation processes, and nonextensivity in some thermodynamic quantities [1,2,3,4]. These systems are usually characterized by a large number of components immersed in random or disordered media that interact through long-range forces and/or possess long time memories; as a consequence, they may present a collective behavior very different from those of their individual components. Many of the above-mentioned phenomena have been understood appropriately by means of proposals of generalized entropies [4,5,6,7,8,9,10,11,12], which have found grounds on diverse applications within the realm of complex systems (see, e.g., Ref. [9] for a comprehensive list of entropic forms available in the literature up to 2011). In its statistical formulation, a given entropic form should be a functional only of a set $\left\{P_{i}\left(t\right)\right\}$, i.e., $S\equiv S(\left\{P_{i}\left(t\right)\right\})$, where $P_{i}\left(t\right)$ stands for the probability of finding a given system on a state *i* at time *t* [13,14]. Most of these generalized entropies violate the additivity property and are usually referred to as non-additive entropic forms. This property concerns two probabilistically independent systems (*A* and *B*), described by two sets of probabilities $\left\{P_{i}^{\left(A\right)}\right\}$ and $\left\{P_{j}^{\left(B\right)}\right\}$, respectively, such that the probabilities for the composed system are given by $P_{ij}^{\left(A+B\right)}=P_{i}^{\left(A\right)}P_{j}^{\left(B\right)}$ (∀(i,j)). A given entropic form is considered non-additive if

(1) $$S^{\left(A+B\right)}\left(\left\{P_{ij}\right\}\right)\neq S^{\left(A\right)}\left(\left\{P_{i}\right\}\right)+S^{\left(B\right)}\left(\left\{P_{j}\right\}\right)\;.$$

Among the many proposals of generalized (or non-additive) entropies, the most commonly known is Tsallis entropy $S_{q}$ [12], which is characterized by an index *q* (q∈R),

(2) $$S_{q}\left(\left\{P_{i}\right\}\right)=k\;\frac{1-\sum_{i=1}^{W}P_{i}^{q}}{q-1}\;,$$

so as to recover the Boltzmann–Gibbs (BG) entropy,

(3) $$S_{\mathrm{BG}}\left(\left\{P_{i}\right\}\right)=-k\sum_{i}P_{i}\left(t\right)\ln P_{i}\left(t\right)\;,$$

in the limit q→1, i.e., $S_{1}\equiv S_{\mathrm{BG}}$.

One of the most successful theories of contemporary theoretical physics is BG statistical mechanics [13,14,15,16,17]; this theory is based on BG entropy, which is additive. The time evolution of $S_{\mathrm{BG}}\left(\left\{P_{i}\left(t\right)\right\}\right)$, and consequently, its approach to the equilibrium state, is directly related to the evolution of the probabilities $\left\{P_{i}\left(t\right)\right\}$, which follow some fundamental equation, e.g., a master equation. For continuous probability densities $P(\vec{x},t)$, the linear Fokker–Planck equation (FPE) appears to be an appropriate candidate for describing the evolution of probabilities and represents one of the most important equations of nonequilibrium BG statistical mechanics. The FPE delineates the time evolution of the probability density $P(\vec{x},t)$ for finding a given particle at a position $\vec{x}$ at time *t* while diffusing under an external potential [14,15,16,17,18]. Usually one considers a confining external potential, leading to the possibility of a stationary-state solution after a sufficiently long time. Particular interest in the literature has been given to a harmonic confining potential, which leads to a Gaussian distribution as the stationary-state solution of the FPE [17,18]. In the absence of an external potential, the FPE reduces to the linear diffusion equation, which does not present a stationary-state solution and is also associated with many out-of-equilibrium applications, such as the celebrated Brownian motion and related phenomena.

A clear understanding of the range of applicability of BG statistical mechanics has been emerging in the latest years; for example, it has become evident that it should be used for systems characterized by weakly interacting particles and/or short time memories. As typical counter-examples, regarding diffusion, it is very frequent nowadays to find dynamical behavior that falls out of the ambit of the linear cases, which are commonly called anomalous diffusion and usually take place in media presenting randomness, porosity, and heterogeneity [19]. To deal with these phenomena, one habitually uses a nonlinear (power-like) diffusion equation, known in the literature as a porous media equation [20]. Similar to the linear FPE, by adding a confining potential contribution, one obtains a nonlinear Fokker–Planck equation (NLFPE) [21], as introduced in Refs. [22,23]. For a harmonic confining potential, this NLFPE presents a *q*-Gaussian distribution typical of nonextensive statistical mechanics [4,5,6] as its stationary-state solution. This distribution is expressed as

(4) $$P_{q}\left(u\right)=P_{0}\exp_{q}\left(-\beta u^{2}\right)\;$$

and can be defined in terms of the *q*-exponential function,

(5) $$\exp_{q}\left(u\right)={\left[1+\left(1-q\right)u\right]}_{+}^{1/(1-q)}\;;\;\left(\exp_{1}\left(u\right)=\exp\left(u\right)\right)\;,$$

where $P_{0}\equiv P_{q}\left(0\right)$ and ${\left[y\right]}_{+}=y$ for y>0 (zero otherwise). In this way, the NLFPE introduced in Refs. [22,23] is associated with the Tsallis entropy, $S_{q}$, since its *q*-Gaussian solution coincides with the distribution that maximizes $S_{q}$. Additionally, proofs of an H-theorem connect the linear FPE with BG entropy [17,18], as well as NLFPEs with generalized entropies [21,24,25,26,27,28,29,30,31,32,33,34,35], and particularly, they relate the NLFPE of Refs. [22,23] to the entropy $S_{q}$.

In the present work, we analyze general entropic forms (typically non-additive) for both continuous and discrete probabilities whose time evolution follows a NLFPE, or a master equation, respectively. Some important novel results from the thermodynamical point of view, related to their corresponding equilibrium states, are studied. In the next section, we define general NLFPEs and explore their relationship to non-additive entropies by means of an H-theorem. Additionally, the corresponding stationary-state solutions are discussed; due to the H-theorem, after a sufficiently long time, the system should reach an equilibrium state for which a given stationary-state solution holds as the equilibrium solution. At equilibrium, we show that the Carnot cycle applies for these entropic forms under very common conditions. In Section 3, we consider the case of discrete probabilities, whose time evolution follows a master equation, while also proving an H-theorem; moreover, the third law of thermodynamics is discussed for both $S_{q}$ and general entropic forms. Finally, in Section 4, we present our main conclusions.

## 2. Continuous Probabilities: Non-Additive Entropic Forms and NLFPEs

Although one may pursue an analysis in arbitrary dimensions, by considering a probability density $P(x_{1},x_{2},\cdots ,x_{N},t)$ (such as, e.g., in Ref. [34]) herein for simplicity, we will restrict ourselves to a one-dimensional space described in terms of a probability density P(x,t) and following the normalization condition

(6) $$\int_{-\infty }^{\infty }P\left(x,t\right)dx=1\;.$$

In this case, a general NLFPE may be defined as [30,31]

(7) $$\frac{\partial P(x,t)}{\partial t}=-\frac{\partial }{\partial x}\left\{A\left(x\right)\Psi \left[P\left(x,t\right)\right]\right\}+D\frac{\partial }{\partial x}\left\{\Omega \left[P\left(x,t\right)\right]\frac{\partial P(x,t)}{\partial x}\right\}\;,$$

where *D* represents a diffusion coefficient with dimensions of energy, and the external force A(x) is associated with a confining potential ϕ(x) [A(x)=−dϕ(x)/dx]. The functionals Ψ[P(x,t)] and Ω[P(x,t)] should satisfy certain mathematical requirements, e.g., positiveness and monotonicity with respect to P(x,t) [30,31]; moreover, to ensure the normalizability of P(x,t) for all times, one must impose the conditions

(8) $${P\left(x,t\right)|}_{x\to \pm \infty }=0\;;\;{\left.\frac{\partial P(x,t)}{\partial x}\right|}_{x\to \pm \infty }{=0\;;\;A\left(x\right)\Psi \left[P\left(x,t\right)\right]|}_{x\to \pm \infty }=0\;\left(\forall t\right)\;.$$

The NLFPE of Equation (7) recovers some well-known cases as particular limits: (i) the linear FPE [14,15,16,17,18] for Ψ[P(x,t)]=P(x,t) and Ω[P(x,t)]=1 and (ii) the NLFPE introduced in Refs. [22,23], which are associated with nonextensive statistical mechanics, for Ψ[P(x,t)]=P(x,t) and $\Omega \left[P\left(x,t\right)\right]=\mu {\left[P\left(x,t\right)\right]}^{\mu -1}$, where μ represents a real number related to the entropic index through μ=2−q. It should be mentioned that a large variety of NLFPEs, such as the one related to nonextensive statistical mechanics, the one in the general form of Equation (7), or even those presenting nonhomogeneous diffusion coefficients in the nonlinear diffusion term, have been derived in the literature by generalizing standard procedures applied to the linear FPE [14,15,16,17,18], e.g., from approximations in the master equation [34,36,37,38,39] or from a Langevin approach considering a multiplicative noise [40,41,42,43,44,45].

Almost two decades ago, NLFPEs presenting more than one diffusive term appeared in the literature [36,46,47,48,49,50,51,52], and a special interest was given to a concrete physical application, namely, a system of interacting vortices, which is currently used as a suitable model for type II superconductors, that exhibited such a behavior [47,48,49,50,51,52]. A general discussion of NLFPEs with two diffusive contributions was presented in Ref. [33], where one can be identified in Equation (7),

(9) $$\Omega \left[P\left(x,t\right)\right]=\frac{D_{1}}{D}\;\Omega_{1}\left[P\left(x,t\right)\right]+\frac{D_{2}}{D}\;\Omega_{2}\left[P\left(x,t\right)\right]\;.$$

Next, we discuss the H-theorem associated with Equation (7), leading to a direct connection between this equation and entropic forms; we also comment on the above case of two diffusive contributions.

### 2.1. Generalized Forms of the H-Theorem from NLFPEs

The H-theorem represents one of the most important results of nonequilibrium statistical mechanics since it ensures that after a sufficiently long time, the associated system will reach an equilibrium state. In standard nonequilibrium statistical mechanics, it is usually proven by considering the BG entropy $S_{\mathrm{BG}}$ and making use of an equation that describes the time evolution of the associated probability density, such as the Boltzmann probability density, linear FPE (in the case of continuous probabilities), or the master equation (in the case of discrete probabilities) [13,14,15,16,17]. To our knowledge, the first proof of an H-theorem making use of a NLFPE appeared in the literature more than 30 years ago [53]. After that, proofs were extended by many authors in such a way as to cover generalized entropic forms and their relationships to NLFPEs (see, e.g., Refs. [21,24,25,26,27,28,29,30,31,32,33,34,35]); below, we closely follow those carried in Refs. [30,31,32,33].

In the case of a system under a confining external potential ϕ(x) (from which one obtains the external force appearing in Equation (7), A(x)=−dϕ(x)/dx), the H-theorem corresponds to a well-defined sign for the time derivative of the free-energy functional,

(10) $$F\left[P\right]=U\left[P\right]-\theta S\left[P\right]\;;\;U\left[P\right]=\int_{-\infty }^{\infty }\;dx\;\phi \left(x\right)P\left(x,t\right)\;,$$

with θ denoting a positive parameter with dimensions of temperature. Moreover, the entropy may be considered in the general form [30,31,32,33],

(11) $$S\left[P\right]=k\int_{-\infty }^{\infty }\;dx\;g\left[P\left(x,t\right)\right]\;;\;g\left(0\right)=g\left(1\right)=0\;;\;\frac{d^{2}g}{dP^{2}}\leq 0\;,$$

where *k* represents a positive constant with entropy dimensions, whereas the functional g[P(x,t)] should be at least twice differentiable. Furthermore, the conditions that ensure the normalizability of P(x,t) for all times (cf. Equation (8)) are also used in the proof of the H-theorem. Considering D=kθ, the H-theorem may be achieved by imposing the condition [30,31,32,33],

(12) $$-\frac{d^{2}g\left[P\right]}{dP^{2}}=\frac{\Omega [P]}{\Psi [P]}\;,$$

which relates the entropic form to a certain time evolution described by the two functionals of Equation (7). Particular entropic forms and their associated NLFPEs were explored in Ref. [30], whereas families of NLFPEs (those characterized by the same ratio Ω[P]/Ψ[P]) were studied in Ref. [32].

One should mention that the relationship of Equation (12) is applicable for a single diffusive contribution, a linear internal-energy definition (as in Equation (10)), and for a constant diffusion coefficient, as in Equation (7). Extensions of the H-theorem have been achieved, disregarding these restrictions separately, by considering: (i) two diffusive contributions, as in Equation (9) [33] (to be discussed next); (ii) a nonlinear internal-energy definition (see Refs. [31,34]). In this case, the H-theorem is fulfilled through a slight modification in the NLFPE of Equation (7), so that besides Equation (12), an extra equation appears concerning the nonlinear functional appearing in the internal energy definition; (iii) a diffusion coefficient dependent on the position, so that one needs to modify the free energy of Equation (10) [54]. Recent studies have discussed physical systems within the context of nonextensive statistical mechanics, characterized by a varying entropic index *q*, such as a modified cosmological scenario [55], and the phenomenon of quantum mixing, i.e., the superposition of particle states with different masses [56]. This is certainly an interesting novelty, not contemplated by Equation (7), which recovers the NLFPE introduced in Refs. [22,23] and is associated with nonextensive statistical mechanics for Ψ[P(x,t)]=P(x,t) and $\Omega \left[P\left(x,t\right)\right]=\left(2-q\right){\left[P\left(x,t\right)\right]}^{1-q}$, where *q* represents a real number. The solution of this NLFPE is the so-called *q*-Gaussian distribution (cf. Equation (4)), which was shown to cover a large number of experimental verifications within the context of anomalous diffusion phenomena, for which the value of *q* may vary for different systems [4,5,6,7,8]. An NLFPE with a variable index *q*, its solution, as well as a possible H-theorem, require a particular nontrivial analysis, which, to our knowledge, has not been addressed at present.

A detailed proof of the H-theorem in the case of two diffusive contributions, such as in Equation (9), was presented in Ref. [33]; briefly, one replaces the free energy functional of Equation (10) with

(13) $$F\left[P\right]=U\left[P\right]-\theta_{1}S_{1}\left[P\right]-\theta_{2}S_{2}\left[P\right]\;;\;U\left[P\right]=\int_{-\infty }^{\infty }\;dx\;\phi \left(x\right)P\left(x,t\right)\;,$$

where $\theta_{1}$ and $\theta_{2}$ denote positive parameters with dimensions of temperature. Similarly to Equation (11), one defines

(14) $$S_{i}\left[P\right]=k\int_{-\infty }^{\infty }\;dx\;g_{i}\left[P\left(x,t\right)\right]\;;\;g_{i}\left(0\right)=g_{i}\left(1\right)=0\;;\;\frac{d^{2}g_{i}}{dP^{2}}\leq 0\;;\;\left(i=1,2\right).$$

In such a case, it is sufficient to impose the conditions

(15) $$D_{1}=k\theta_{1}\;;\;\;D_{2}=k\theta_{2}\;,$$

as well as

(16) $$-\frac{d^{2}g_{1}\left[P\right]}{dP^{2}}=\frac{\Omega_{1}\left[P\right]}{\Psi [P]}\;;\;\;-\frac{d^{2}g_{2}\left[P\right]}{dP^{2}}=\frac{\Omega_{2}\left[P\right]}{\Psi [P]}\;,$$

extending the condition of Equation (12) for two diffusion contributions.

From now on, we restrict our analysis to a single diffusion contribution, as in Equation (7), and their associated free energy functional (cf. Equation (10)), entropy functional (cf. Equation (11)), as well as the relationship in Equation (12). In the discussion above, this situation occurs whenever a diffusion coefficient is much larger than the other one (e.g., $D_{2}\gg D_{1}$) so that one may neglect the effects of the smaller contribution. As a typical example, one should mention a system of interacting vortices currently used as a suitable model for type II superconductors, for which, in typical cases, one of the diffusion coefficients has been shown to be at least $10^{4}$ times larger than the other one [49].

### 2.2. Equilibrium Distribution

Now, we briefly work out the stationary-state (i.e., time-independent) solution of Equation (7), as well as the equilibrium distribution that results from an extremization procedure of the entropic functional in Equation (11) (a detailed analysis of these procedures may be found in Ref. [30])). As usual, the Lagrange parameters of this later approach will be defined appropriately so that these two results coincide; based on this, in the calculations that follow, we refer to an equilibrium state, described by a distribution $P_{\mathrm{eq}}\left(x\right)$.

First, let us obtain the time-independent distribution of Equation (7); for this purpose, we rewrite it in the form of a continuity equation,

(17) $$\frac{\partial P(x,t)}{\partial t}=-\frac{\partial J(x,t)}{\partial x}\;,$$

where the probability current density is given by

(18) $$J\left(x,t\right)=A\left(x\right)\Psi \left[P\left(x,t\right)\right]-D\Omega \left[P\left(x,t\right)\right]\frac{\partial P(x,t)}{\partial x}\;.$$

The solution $P_{\mathrm{eq}}\left(x\right)$ is obtained by setting $J_{\mathrm{eq}}\left(x\right)=0$ (as required by conservation of probability [30]), so that

(19) $$J_{\mathrm{eq}}\left(x\right)=A\left(x\right)\Psi \left[P_{\mathrm{eq}}\left(x\right)\right]-D\Omega \left[P_{\mathrm{eq}}\left(x\right)\right]\frac{dP_{\mathrm{eq}}}{dx}=0\;,$$

which may still be written in the form

(20) $$A\left(x\right)=D\;\frac{\Omega [P_{\mathrm{eq}}\left(x\right)]}{\Psi [P_{\mathrm{eq}}\left(x\right)]}\frac{dP_{\mathrm{eq}}}{dx}\;.$$

Integrating the equation above over *x* and remembering that the external force was defined as A(x)=−dϕ(x)/dx, one obtains

(21) $$\phi_{0}-\phi \left(x\right)=D\int_{x_{0}}^{x}dx\;\frac{\Omega [P_{\mathrm{eq}}\left(x\right)]}{\Psi [P_{\mathrm{eq}}\left(x\right)]}\frac{dP_{\mathrm{eq}}}{dx}=D\int_{P_{\mathrm{eq}}\left(x_{0}\right)}^{P_{\mathrm{eq}}\left(x\right)}\;\frac{\Omega [P_{\mathrm{eq}}\left(x^{\prime }\right)]}{\Psi [P_{\mathrm{eq}}\left(x^{\prime }\right)]}dP_{\mathrm{eq}}\left(x^{\prime }\right)\;,$$

where $\phi_{0}\equiv \phi \left(x_{0}\right)$. Now, one uses the relationship in Equation (12), and, performing the integration, can further obtain

(22) $${\left.D\frac{dg[P]}{dP}\right|}_{P=P_{\mathrm{eq}}\left(x\right)}=\phi \left(x\right)+C_{1}\;,$$

with $C_{1}$ being a constant.

Next, we extremize the entropic functional of Equation (11) with respect to the probability under the constraints of probability normalization and an internal energy definition following Equation (10). For this, we introduce the functional

(23) $$I=\frac{S[P]}{k}+\alpha \left(1-\int_{-\infty }^{\infty }dx\;P\left(x,t\right)\right)+\beta \left(U-\int_{-\infty }^{\infty }dx\;\phi \left(x\right)P\left(x,t\right)\right)\;,$$

where α and β are Lagrange multipliers. Hence, the extremization ${\left(\delta I\right)/\left(\delta P\right)|}_{P=P_{\mathrm{eq}}\left(x\right)}=0$ leads to

(24) $${\left.\frac{dg[P]}{dP}\right|}_{P=P_{\mathrm{eq}}\left(x\right)}-\alpha -\beta \;\phi \left(x\right)=0\;.$$

One notices that Equations (22) and (24), which result from the stationary-state solution of Equation (7) and the extremization of the entropic functional of Equation (11), respectively, coincide if one chooses the Lagrange multipliers $\alpha =C_{1}$ and β=1/D.

### 2.3. Carnot Cycle for a General Entropic Form S(P)

Considering non-additive entropies, the Carnot cycle was shown to hold for the equilibrium entropy $S_{2-q}$ (in the case that q=0) and its corresponding thermodynamically conjugated parameter θ [49], which is used to define an infinitesimal heat-like quantity $\delta Q=\theta dS_{2}$ [50,51,52]; the physical system under investigation was a model for type II superconductors characterized by interacting vortices. Later on, the Carnot cycle was shown to be valid for any system of particles interacting repulsively through short-range potentials, whose equilibrium distributions are compact *q*-Gaussian distributions (characterized by a cutoff) and can be described by the entropy $S_{2-q}$ (for q<1), extending the above-mentioned proof for q=0 [57]. One should notice that, in the illustrations concerning the Tsallis entropy considered herein, the equilibrium distribution and the entropic form are related by means of the simple change q↔(2−q) [58]. This appears to be a direct consequence of a linear internal energy definition, such as the one in Equation (10), which was considered in Refs. [50,51,52,57]; this subtle property will be discussed in detail for the case of discrete probabilities (see the next section). Herein, we show that the Carnot cycle holds for general entropic forms, as defined in Equation (11). For this, we assume that the usual (i.e., very common) conditions apply for the system under investigation, as described below.

(i) The equilibrium distribution $P_{\mathrm{eq}}\left(x\right)$, which maximizes the entropic functional of Equation (11) (as shown in Section 2.2), exists and leads to the entropy $S[P_{\mathrm{eq}}]$ and internal energy $U[P_{\mathrm{eq}}]$ at equilibrium. Both $S[P_{\mathrm{eq}}]$ and $U[P_{\mathrm{eq}}]$ are state functions in the sense that

(25) $$\begin{array}{ccc} \int_{a}^{b}dS\left[P_{\mathrm{eq}}\right] & = & S\left[P_{\mathrm{eq}}^{\left(b\right)}\right]-S\left[P_{\mathrm{eq}}^{\left(a\right)}\right]=S_{b}-S_{a}\;; \end{array}$$

(26) $$\begin{array}{ccc} \int_{a}^{b}dU\left[P_{\mathrm{eq}}\right] & = & U\left[P_{\mathrm{eq}}^{\left(b\right)}\right]-U\left[P_{\mathrm{eq}}^{\left(a\right)}\right]=U_{b}-U_{a}\;, \end{array}$$

where *a* and *b* denote arbitrary equilibrium thermodynamic states. We introduce the short notations $S_{a}\equiv S\left[P_{\mathrm{eq}}^{\left(a\right)}\right]$ and $U_{a}\equiv U\left[P_{\mathrm{eq}}^{\left(a\right)}\right]$ (similar notations holding for state *b*). Hence, one may define an infinitesimal type of heat, δQ=θdS, where θ represents the positive parameter with the temperature dimensions introduced in Equation (10).

(ii) The system under investigation can, in principle, perform work in several ways, leading to an infinitesimal contribution, $\delta W=\sum_{i}\sigma_{i}d\alpha_{i}$, where for each contribution *i*, $\sigma_{i}$ and $\alpha_{i}$ are pairs of thermodynamically conjugate variables. However, for simplicity, we restrict the following analysis to a single “external field”, σ, and its conjugate, α. The parameter α is also considered a state function following conditions similar to those in Equations (25) and (26).

(iii) Using the quantities defined in (i) and (ii), we formulate the equivalent to the first law,

(27) dU=δQ+δW=θdS+σdα,

where δW corresponds to the work carried out by the external field σ *on* the system.

(iv) Equation (27) implies that U=U(S,α); we assume that U(S,α) is invertible, yielding S=S(U,α) (with the same condition holding for S(U,α)), leading to

(28) $$dS=\frac{1}{\theta }dU-\frac{\sigma }{\theta }d\alpha \;.$$

From Equation (27) (or equivalently, from Equation (28)) one obtains the fundamental relationship

(29) $$\theta ={\left(\frac{\partial U}{\partial S}\right)}_{\alpha }\;,$$

as well as the equation of state

(30) $$\sigma ={\left(\frac{\partial U}{\partial \alpha }\right)}_{S}\;.$$

Let us now consider four equilibrium states, yielding a Carnot cycle a→b→c→d→a, defined by two isothermal (constant θ) transformations: a→b at a temperature $\theta_{1}$ and c→d at a temperature $\theta_{2}$, with $\theta_{1}>\theta_{2}$. These transformations are intercalated by two adiabatic transformations (where *S* is constant) (b→c and d→a), so that $\Delta S_{bc}=\Delta S_{da}=0$. Considering that both *S* and *U* are state functions (according to Equations (25) and (26)), and using Equation (27), one has for the whole cycle

(31) $$\begin{array}{ccc} \Delta S & = & \Delta S_{ab}+\Delta S_{cd}=0\;\Rightarrow \;\Delta S_{ab}=-\Delta S_{cd}\;; \end{array}$$

(32) $$\begin{array}{ccc} \Delta U & = & \left(W_{ab}+Q_{ab}\right)+W_{bc}+\left(W_{cd}+Q_{cd}\right)+W_{da}=0\;. \end{array}$$

From this later equation, one can obtain that the total work *W* carried out *on* the system is given by

(33) $$W=W_{ab}+W_{bc}+W_{cd}+W_{da}=-\left(Q_{ab}+Q_{cd}\right)=-W\;,$$

where we have defined W (W>0) as the total work completed *by* the system. For the two isothermal transformations, one has

(34) $$\begin{array}{ccc} Q_{ab} & = & \theta_{1}\int_{a}^{b}dS=\theta_{1}\left(S_{b}-S_{a}\right)=\theta_{1}\Delta S_{ab}\;; \end{array}$$

(35) $$\begin{array}{ccc} Q_{cd} & = & \theta_{2}\int_{c}^{d}dS=\theta_{2}\left(S_{d}-S_{c}\right)=\theta_{2}\Delta S_{cd}\;, \end{array}$$

and using Equation (31), one obtains that

(36) $$\frac{Q_{ab}}{Q_{cd}}=-\frac{\theta_{1}}{\theta_{2}}\;,$$

showing that $Q_{ab}$ and $Q_{cd}$ present different signs. Therefore, as usually considered for a Carnot Cycle, we assume that $Q_{ab}>0$ and $Q_{cd}<0$, i.e., heat gets into (out of) the system along the isothermal transformation at temperature $\theta_{1}$ ($\theta_{2}$). Let us now redefine $Q_{1}=Q_{ab}$ and $Q_{2}=\left|Q_{cd}\right|$, leading to the fundamental relationship for the Carnot Cycle,

(37) $$\frac{Q_{1}}{Q_{2}}=\frac{\theta_{1}}{\theta_{2}}\;,$$

as well as to the conservation of energy along the whole cycle, which can be expressed as

(38) $$Q_{1}=W+Q_{2}\;,$$

Tn these two equations above, all quantities are positive. Consequently, one has the celebrated efficiency for the Carnot cycle,

(39) $$\eta =\frac{W}{Q_{1}}=\frac{Q_{1}-Q_{2}}{Q_{1}}=1-\frac{\theta_{2}}{\theta_{1}}\;\left(0\leq \eta \leq 1\right).$$

Therefore, we have shown that the Carnot cycle, which represents a fundamental thermodynamical process, holds for general entropic forms as defined in Equation (11) and for the internal energy of Equation (10) under the usual requirements for its equilibrium state. Within the framework of non-additive entropies, the most serious restrictions are: (a) at equilibrium, $S\equiv S[P_{\mathrm{eq}}]$ and $U\equiv U[P_{\mathrm{eq}}]$, so that one must express $S=S(U,\left\{\alpha_{i}\right\})$, where $\left\{\alpha_{i}\right\}$ represents state functions, whose small changes define infinitesimal work contributions; (b) the entropy $S=S(U,\left\{\alpha_{i}\right\})$ should be invertible, leading to the possibility of expressing $U=U(S,\left\{\alpha_{i}\right\})$. Only if these conditions are satisfied may one be able to calculate an effective temperature in two different (but equivalent) ways,

(40) $$\theta ={\left(\frac{\partial U}{\partial S}\right)}_{\left\{\alpha_{i}\right\}}\;\mathrm{and}\;\frac{1}{\theta }={\left(\frac{\partial S}{\partial U}\right)}_{\left\{\alpha_{i}\right\}}\;,$$

using Equations (27) and (28), respectively.

To our knowledge, at present, the only successful proofs of a Carnot cycle for non-additive entropies have been carried for the equilibrium entropy $S_{2-q}$ (in the case that q=0) and its corresponding thermodynamically conjugate parameter θ in an application of a system of type II superconducting vortices [49,50,51,52], as well as an extension for the equilibrium entropy $S_{2-q}$ (for q<1) associated with a system of particles interacting repulsively through short-range potentials, whose equilibrium distributions are compact *q*-Gaussian distributions (characterized by a cutoff) [57]. The proof above opens the way for the validation of the Carnot cycle considering other non-additive entropic forms available in the literature.

## 3. Discrete Set of Probabilities: H-Theorem and Equilibrium Solutions for Generalized Entropies

We now consider a system characterized by discrete states, with associated probabilities $\left\{P_{i}\left(t\right)\right\}$ (i=1,2,⋯,W), where $P_{i}\left(t\right)$ represents the probability of finding the system on a state *i* at time *t*, following the normalization condition

(41) $$\sum_{i=1}^{W}P_{i}\left(t\right)=1\;\left(\forall t\right).$$

For discrete probabilities, an H-theorem was proven for $S_{\mathrm{BG}}\left(\left\{P_{i}\right\}\right)$ (cf. Equation (3)) in both cases of an isolated system (expressed by $dS_{\mathrm{BG}}/dt\geq 0$) and a system in contact with a heat bath at a temperature *T*, where one can consider the time-derivative of the free-energy functional to be

(42) $$F=U-TS_{\mathrm{BG}}\;;\;\;U=\sum_{i}\varepsilon_{i}P_{i}\;,$$

leading to dF/dt≤0 (see, e.g., Ref. [13]).

Recently, there has been a growing interest in generalized entropic forms for an appropriate description of complex systems [4,5,6,7,8,9,10,11]. In most cases, these entropic forms may be written as

(43) $$S\left[\left\{P_{i}\right\}\right]=k\sum_{i=1}^{W}g\left[P_{i}\right]\;;\;g\left(0\right)=g\left(1\right)=0\;,$$

where the functional $g[P_{i}]$ should be concave and at least twice-differentiable, i.e., $\left(d^{2}g/d{P_{i}}^{2}\right)\leq 0\;\left(\forall i\right)$. Moreover, the free-energy functional is considered similar to the one in Equation (42),

(44) $$F=U-\theta S\;;\;\;U=\sum_{i}\varepsilon_{i}P_{i}\;,$$

where θ represents a positive quantity with dimensions of temperature, which in some cases may coincide with the usual absolute temperature *T*, although it may present a different concept in some complex systems (see, e.g., Refs. [49,50,51,52]).

The extremization of the entropic form of Equation (43), considering the constraints for the probability normalization of Equation (41) (with the Lagrange multiplier α) and the internal energy definition of Equation (44) (with its corresponding Lagrange multiplier β), leads to the following equation for the equilibrium distribution $P_{i}^{\mathrm{eq}}$:

(45) $$g^{\prime }\left[P_{i}^{\mathrm{eq}}\right]-\alpha -\beta \varepsilon_{i}=0\;,$$

where we have defined

(46) $$g^{\prime }\left(X\right)\equiv {\left.\frac{dg[P]}{dP}\right|}_{P=X}\;.$$

One should notice that the functional $g^{\prime }\left[P_{i}^{\mathrm{eq}}\right]$ is invertible, since $g[P_{i}]$ is concave; however, in some cases, one may deal with a transcendental equation for $P_{i}^{\mathrm{eq}}$. The procedure above applied to BG entropy (cf. Equation (3)) yields the well-known Boltzmann weight [13,14]; let us now illustrate this method by considering the Tsallis entropy (cf. Equation (2)), for which

(47) $$g^{\prime }\left[P_{i}^{\mathrm{eq}}\right]=-\frac{q}{q-1}{\left(P_{i}^{\mathrm{eq}}\right)}^{q-1}\;.$$

Substituting the result above into Equation (45) and using Equation (41), one obtains the distribution

(48) $$P_{i}^{\mathrm{Equation}\;(1)}=\frac{1}{Z_{q}^{\left(1\right)}}{\left[1-\left(q-1\right)\beta^{\left(1\right)}\varepsilon_{i}\right]}_{+}^{1/(q-1)}\;,$$

where ${\left[y\right]}_{+}=y$ for y>0 and is zero otherwise; the superscript refers to the extremization of the entropy $S_{q}$ under the internal energy definition with a linear dependence on the set of probabilities, as in Equation (44), which is also known as first formulation [4]. It is important to notice that the equilibrium distribution coming out of the extremization procedure of any entropic form is directly related to the imposed constraints (see, e.g., Refs. [58,59,60,61,62] for detailed discussions on the role of constraints in nonextensive statistical mechanics); herein, we adopt the linear internal energy definition due to its simplicity for proving the H-theorem. However, the most common form for the equilibrium distribution, usually known as the Tsallis distribribution, is obtained from an internal energy definition with a nonlinear dependence on the set of probabilities $\left\{P_{i}\right\}$, i.e., a power-like $P_{i}^{q}$, leading to [4]

(49) $$P_{i}^{\mathrm{eq}}=\frac{1}{Z_{q}}{\left[1-\left(1-q\right)\beta \varepsilon_{i}\right]}_{+}^{1/(1-q)}\;,$$

which will be considered the equilibrium distribution from now on. Notice that Equations (48) and (49) may be converted into one another by means of the simple change q↔(2−q) [58]. In fact, the distribution of Equation (49) may also be derived from the extremization of $S_{2-q}$ in Equation (45). Since in the thermodynamic application that follows, namely, the third law of thermodynamics for the Tsallis entropy, we consider an equilibrium state described by Equation (49), the corresponding entropic form can be written as $S_{2-q}$ instead of $S_{q}$.

Below, we outline the proof of an H-theorem for general entropic forms, written as in Equation (43), which may be achieved by making use of a master equation [63],

(50) $$\frac{\partial P_{i}\left(t\right)}{\partial t}=\sum_{j}\left[P_{j}\left(t\right)w_{ji}\left(t\right)-P_{i}\left(t\right)w_{ij}\left(t\right)\right]\;.$$

As usual, $w_{ij}\left(t\right)$ represents the probability transition rate associated with a transition from state *i* to state *j* (i.e., $w_{ij}\Delta t$ is the probability that a transition from states *i* to *j* occurs during the time interval t→t+Δt). Herein, we will consider the most general out-of-equilibrium situation characterized by time-dependent probability transition rates, i.e., $P_{i}\left(t\right)w_{ij}\left(t\right)\neq P_{j}\left(t\right)w_{ji}\left(t\right)$. These quantities will become time-independent only at equilibrium, where the detailed balance condition holds,

(51) $$P_{i}^{\mathrm{eq}}W_{ij}=P_{j}^{\mathrm{eq}}W_{ji}\;\;\left[W_{ij}=\lim_{t\to \infty }w_{ij}\left(t\right)\right]\;.$$

The procedure below essentially extends the proof of the H-theorem for BG entropy in Equation (3) for an isolated system, as well as for a system in contact with a heat bath at a temperature *T*, where one considers the time derivative of the free-energy functional in Equation (42) (see, e.g., Ref. [13]). Following this, we start by taking the time derivative of the entropic form in Equation (43),

(52) $$\frac{dS}{dt}=k\frac{d}{dt}\sum_{i}g\left[P_{i}\left(t\right)\right]=k\sum_{i}\frac{dg}{dP_{i}}\frac{\partial P_{i}}{\partial t}\;,$$

and using Equation (50), one obtains

(53) $$\frac{dS}{dt}=k\sum_{i,j}\frac{dg}{dP_{i}}\left[P_{j}\left(t\right)w_{ji}\left(t\right)-P_{i}\left(t\right)w_{ij}\left(t\right)\right]\;.$$

Interchanging i↔j and adding the resulting equation with Equation (53), one obtains

(54) $$\frac{dS}{dt}=\frac{k}{2}\sum_{i,j}\left[g^{\prime }\left(P_{i}\right)-g^{\prime }\left(P_{j}\right)\right]\left[P_{j}\left(t\right)w_{ji}\left(t\right)-P_{i}\left(t\right)w_{ij}\left(t\right)\right]\;,$$

where we have used the definition of Equation (46). In a similar way, one can express the time derivative of the internal energy of Equation (44) as

(55) $$\frac{dU}{dt}=\frac{1}{2}\sum_{i,j}\left[\varepsilon_{i}-\varepsilon_{j}\right]\left[P_{j}\left(t\right)w_{ji}\left(t\right)-P_{i}\left(t\right)w_{ij}\left(t\right)\right]\;.$$

Now, using Equations (44), (54) and (55), one obtains the time derivative of the free-energy functional,

(56) $$\frac{dF}{dt}=\frac{1}{2}\sum_{i,j}\left\{\varepsilon_{i}-\varepsilon_{j}-k\theta \left[g^{\prime }\left(P_{i}\right)-g^{\prime }\left(P_{j}\right)\right]\right\}\left[P_{j}\left(t\right)w_{ji}\left(t\right)-P_{i}\left(t\right)w_{ij}\left(t\right)\right]\;.$$

General proofs of the H-theorem have been carried out in Ref. [63] through algebraic manipulations of the equations above (e.g., making use of the property that g[X] is concave, leading to a monotonically decreasing first derivative $g^{\prime }\left[X\right]$) for two typical situations, namely, an isolated system (for which the H-theorem is expressed by (dS/dt)≥0) and a system in contact with a thermal bath (for which the H-theorem is expressed by (dF/dt)≤0). Moreover, these proofs apply to very general out-of-equilibrium situations (along which $P_{i}\left(t\right)w_{ij}\left(t\right)\neq P_{j}\left(t\right)w_{ji}\left(t\right)$) and are characterized by non-symmetric probability transition rates ($w_{ij}\left(t\right)\neq w_{ji}\left(t\right)$). Additional interesting results were achieved in Ref. [64], where the quantities above were associated with the phenomenon of entropy production for irreversible processes. Mathematically, this result may be expressed by writing the entropy time rate in the form [16,65,66]

(57) $$\begin{array}{ccc} \frac{d}{dt}S\left[P\right] & = & \Pi -\Phi , \end{array}$$

where one can identify the contributions of the entropy production Π and entropy flux Φ. These two concepts were extended to general entropic forms, making use of general NLFPEs [67] when dealing with continuous probabilities, as well as of a master equation [64] for discrete probabilities. Comparing the quantities above with

(58) $$\begin{array}{c} \frac{dF}{dt}=\frac{dU}{dt}-\theta \;\frac{dS}{dt}\;, \end{array}$$

one identifies

(59) $$\Pi =-\frac{1}{\theta }\frac{dF}{dt}\;;\;\Phi =-\frac{1}{\theta }\frac{dU}{dt}\;,$$

so that, for a non-negative entropy production contribution, the H-theorem implies Π≥0, as expected [16,65,66]. All these results were illustrated for the particular cases of BG (cf. Equation (3)) and Tsallis (cf. Equation (2)) entropies [63,64].

### Third Law of Thermodynamics for Generalized Entropies

Herein, we assume that the following typical conditions apply.

(i) There is a positive temperature-like parameter θ, associated with the Lagrange multiplier β, such that β=1/(kθ). It is important to mention that the following analysis also applies for the absolute temperature *T* of standard thermodynamics.

(ii) There is a discrete non-negative energy spectrum $\left\{\varepsilon_{i}\right\}$, i.e., $\varepsilon_{i}\geq 0$ (i=1,2,⋯,W).

(iii) There is a non-degenerate ground state characterized by the energy $\varepsilon_{1}\geq 0$.

(iv) There is a gap between the energies of the ground and first-excited states, $\Delta =\varepsilon_{2}-\varepsilon_{1}>0$.

(v) As the temperature parameter θ decreases (or equivalently, as β increases), the probabilities $\left\{P_{i}\right\}$ associated with the lowest-energy states become larger.

Next, we illustrate the third law of thermodynamics for the entropy $S_{q}$ of Equation (2) and its corresponding equilibrium distribution in Equation (49). In this case, for convenience, we set $\varepsilon_{1}=0$. Notice that the requirement for real probabilities in Equation (49) implies the condition

(60) $$\left(1-q\right)\beta \varepsilon_{i}\leq 1\;\left(\forall i\right).$$

Whenever the inequality above is violated, one has $P_{i}=0$, i.e., the corresponding energy level may not be occupied. For the third law of thermodynamics applied under the above requirements, two distinct cases should be considered, namely, q≥1 and q<1, as discussed below.

**Case 1:**  q≥1.

The condition in Equation (60) is always fulfilled, so that for β→∞, the system should reach a pure state, characterized by $P_{1}=1$ and leading to $S_{q}\left(1\right)=S_{2-q}\left(1\right)=0$.

**Case 2:** q<1.

The condition in Equation (60) is not always fulfilled, and it may be violated for certain ranges of β, values of *q*, and energies $\varepsilon_{i}$, for which $P_{i}=0$. Now, we concentrate on the two lowest energy values, separated by the gap $\Delta =\varepsilon_{2}-\varepsilon_{1}$, and define a temperature $\theta^{*}$ through $k\theta^{*}=\left(1-q\right)\Delta$. At precisely the effective temperature $\theta^{*}$, only these two energy levels are occupied with the respective probabilities $P_{2}$ and $P_{1}$ such that $P_{1}+P_{2}=1$. Then, for a slightly lower temperature, one has $P_{2}=0$ and $P_{1}=1$, leading to $S_{q}\left(1\right)=S_{2-q}\left(1\right)=0$. Notice that $k\theta^{*}\leq \Delta$ (for 0≤q<1), whereas $k\theta^{*}=\left(1+|q|\right)\Delta$, yielding $k\theta^{*}>\Delta$ (for q<0).

**Figure 1:**
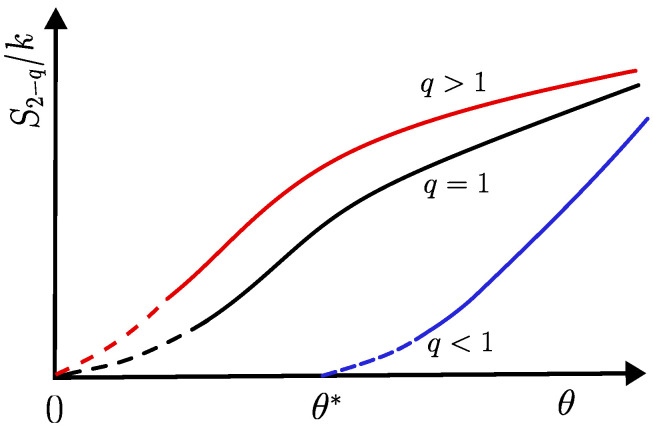
The entropy $S_{2-q}$ is exhibited versus an effective temperature θ (positive quantity with dimensions of temperature) in three distinct cases, namely, q>1 (red curve), q=1 (black curve), and q<1 (blue curve). The entropy $S_{2-q}$ becomes zero for θ→0 (q≥1), whereas $S_{2-q}\to 0$ for $\theta \to \theta^{*}>0$ (q<1) (see text). In the latter case, the vanishing of $S_{2-q}$ at an effective temperature $\theta^{*}>0$ is directly related to violations in the condition of Equation (60), i.e., to the existence of a cutoff in the set of probabilities $\left\{P_{i}\right\}$. In these curves, the approaches $S_{2-q}\to 0$ are shown in dashed lines since the corresponding slopes may depend on the system under study.

Therefore, the entropy $S_{2-q}$ becomes zero for a positive value of temperature, $\theta^{*}=\left(1-q\right)\Delta /k>0$, so that the third law is satisfied at (and below) this temperature value. In this way, the effective temperature $\theta^{*}$ for q<1 plays a role similar to θ=0 for q≥1; thus, in the former case, all thermodynamic quantities should be analyzed in the limit $\theta \to \theta^{*}$ (from above). The vanishing of $S_{2-q}$ at an effective temperature $\theta^{*}>0$ for q<1 is directly related to violations in the condition of Equation (60), i.e., to the existence of a cutoff in the set of probabilities $\left\{P_{i}\right\}$. Qualitative plots of the entropy $S_{2-q}$ versus the effective temperature θ are presented in Figure 1; in each case, the approach to the limits θ→0 (q≥1) or $\theta \to \theta^{*}$ (q<1) are shown in dashed lines since the corresponding slopes may depend on the system under investigation. One should mention that previous studies of the third law for the Tsallis entropy [68,69] did not take into account the effective temperature $\theta^{*}$ for q<1, leading to misinterpretations of the third law of thermodynamics.

Below, we formulate the third law for the entropy $S_{q}$, as well as for nonadditive entropies in general.

**Third law of thermodynamics for the entropy** $S_{q}$:

Consider a system described by: (a) a positive effective temperature θ (for certain values of *q*, this temperature may coincide with the absolute temperature *T* of standard thermodynamics) thermodynamically conjugated to the entropy $S_{q^{*}}$ (typically $q^{*}=2-q$); (b) a discrete non-negative energy spectrum $\left\{\varepsilon_{i}\right\}$; (c) a non-degenerate ground state with energy $\varepsilon_{1}=0$; and (d) a gap between the energies of the ground and the first-excited states, $\Delta =\varepsilon_{2}$. Under these conditions, the entropy $S_{q^{*}}$ becomes zero for θ→0 (q≥1) or for $\theta \to \theta^{*}$ (q<1), where $\theta^{*}=\left(1-q\right)\Delta /k>0$”.

For a general nonadditive entropic form, as defined in Equation (43) and possibly characterized by a set of indices {α,γ,⋯} typical of nonadditive entropic forms [9,10,11], one should add an extra condition in the above statement concerning possible combinations of θ, energy values $\left\{\varepsilon_{i}\right\}$, and the indices {α,γ,⋯}, for which there may be restrictions on the probabilities $\left\{P_{i}\right\}$, such as a cutoff, yielding levels with $P_{i}=0$. In this way, we formulate the third law for a general nonadditive entropy below.

**Third law of thermodynamics for a general nonadditive entropy** $S_{\alpha ,\gamma ,\cdots }$:

Consider a system described by: (a) a positive effective temperature, θ, thermodynamically conjugated to the entropy $S_{\alpha ,\gamma ,\cdots }$ (for certain values of the indices {α,γ,⋯}, this temperature may coincide with the absolute temperature *T* of standard thermodynamics); (b) a discrete non-negative energy spectrum $\left\{\varepsilon_{i}\right\}$; (c) a non-degenerate ground state with energy $\varepsilon_{1}\geq 0$; (d) a gap between the energies of the ground and the first-excited states, $\Delta =\varepsilon_{2}-\varepsilon_{1}>0$; and (e) combinations of θ, the energy values $\left\{\varepsilon_{i}\right\}$, and the indices {α,γ,⋯}, for which there are restrictions on the probabilities $\left\{P_{i}\right\}$, such as a cutoff, yielding levels with $P_{i}=0$. Under these conditions, the entropy $S_{\alpha ,\gamma ,\cdots }$ becomes zero for $\theta \to \theta^{*}$ (with $\theta^{*}>0$), where this threshold should depend on Δ and the parameters {α,γ,⋯} that define the entropy; whenever the restrictions described in (e) do not apply, $S_{\alpha ,\gamma ,\cdots }$ becomes zero for θ→0”.

## 4. Discussion and Conclusions

We have discussed some basic aspects related to non-additive entropies, considering their time evolution in the cases of continuous and discrete probabilities, described by nonlinear Fokker–Planck and master equations, respectively. In both cases, forms of the H-theorem were proven, connecting functionals of the probabilities appearing in these equations with those of the entropic forms. A particular emphasis was given to their equilibrium-state distributions, showing that those obtained from the corresponding evolution equations coincide with those derived form the extremization of the associated entropic forms.

Considering the equilibrium state, we have shown that a Carnot cycle holds for a general entropic form under standard thermodynamic conditions. Within the framework of non-additive entropies, the most serious restrictions for the validity of the Carnot cycle are as follows: (a) the equilibrium functionals $S\equiv S[P_{\mathrm{eq}}]$ and $U\equiv U[P_{\mathrm{eq}}]$ should allow one to express $S=S(U,\left\{\alpha_{i}\right\})$, where $\left\{\alpha_{i}\right\}$ represents state functions, whose small changes define infinitesimal work contributions; (b) the entropy $S=S(U,\left\{\alpha_{i}\right\})$ should be invertible, leading to the possibility of expressing $U=U(S,\left\{\alpha_{i}\right\})$. Only if these conditions are satisfied may one be able to calculate an effective temperature parameter, which is fundamental for the Carnot cycle. It is possible that these procedures may not be feasible for some of the entropic forms introduced in the literature (see, e.g., a comprehensive list in Ref. [9]). To our knowledge, at present, the only successful proofs of a Carnot cycle for non-additive entropies have been carried out for the equilibrium entropy $S_{2-q}$ (in the case for which q=0) and its corresponding thermodynamically conjugate parameter θ in an application of a system of type II superconducting vortices [49,50,51,52], as well as an extension for the equilibrium entropy $S_{2-q}$ (for q<1), associated with a system of particles interacting repulsively through short-range potentials, whose equilibrium distributions are compact *q*-Gaussian distributions (characterized by a cutoff) [57].

In the case of discrete probabilities, we have discussed how the third law of thermodynamics should apply to equilibrium non-additive entropic forms in general. Considering an equilibrium entropic form *S* and its thermodynamically conjugate parameter θ, one has two situations to be analyzed, which are as follows: (i) combinations of θ, energy values $\left\{\varepsilon_{i}\right\}$, and possible entropic indices characteristic of the generalization, for which there are restrictions on the probabilities $\left\{P_{i}\right\}$, such as a cutoff, which implies levels with $P_{i}=0$; (ii) cases where the combinations mentioned in (i) do not lead to restrictions on the probabilities $\left\{P_{i}\right\}$. If there are no restrictions, one must have S→0 when θ→0; whenever there are restrictions on the set of probabilities (such as a possible cutoff), one should have S→0 for $\theta \to \theta^{*}$, where $\theta^{*}>0$.

The physical consequences, and particularly, the fact that the equilibrium-state distributions obtained from the corresponding evolution equations (for both continuous and discrete probabilities) coincide with those obtained from the extremization of the entropic form, become very relevant for the study of complex systems. Moreover, the validity of a Carnot cycle and a formulation of the third law of thermodynamics for general entropic forms, under standard thermodynamic requirements, opens the path for consistent thermodynamic frameworks in the context of generalized (or non-additive) entropic forms.

## Data Availability

Not applicable.
